# Implementation of Ramadan-specific diabetes management recommendations: a multi-centered prospective study from Pakistan

**DOI:** 10.1186/2251-6581-13-37

**Published:** 2014-02-21

**Authors:** Muhammad Yakoob Ahmedani, Syed Faraz Danish Alvi, Muhammad Saif Ul haque, Asher Fawwad, Abdul Basit

**Affiliations:** 1 Department of Medicine, Baqai Institute of Diabetology and Endocrinology, Baqai Medical University, Karachi, Pakistan; 2 Department of Research, Baqai Institute of Diabetology and Endocrinology, Baqai Medical University, Karachi, Pakistan; 3 Department of Biochemistry, Baqai Medical University, Karachi, Pakistan

**Keywords:** Ramadan, Diabetes, Fasting

## Abstract

**Background:**

To observe the outcome of implementation of Ramadan-specific diabetes management recommendations in fasting individuals with diabetes through health care providers.

**Methods:**

This multi-centered prospective study was conducted at nine diabetes specialist centers in four provinces of Pakistan. The study was carried out in two phases; pre-Ramadan recruitment interview (visit A) and post-Ramadan follow up interview (visit B) of the same patients. Pre-Ramadan individual counseling was given and educational material provided to each patient by health care providers during visit A.

**Results:**

Out of 388 patients with diabetes, blood glucose level was checked by all patients with type 1 and 71.43% patients with type 2 diabetes when they developed hypoglycemic symptoms during Ramadan. Of patients with type 1 and type 2 diabetes, 33.33% and 48% discontinued their fast when they felt hypoglycemic symptoms, respectively. None of the patient with type 1, while 18.87% patients with type 2 diabetes discontinued fast on the development of hyperglycemic symptoms. Drug dosage and timing were altered in 80% patients with type 1 and 90.5% patients with type 2 diabetes during Ramadan. Majority of the patients with type 2 diabetes changed from moderate/severe levels of physical activity before Ramadan to light physical activity during Ramadan (p<0.000). None of the patients required hospitalization when they developed symptomatic hypoglycemia or hyperglycemia and none developed diabetic ketoacidosis and hyperglycemic hyperosmolar state during Ramadan.

**Conclusion:**

We observed that it is practicable to implement Ramadan-specific diabetes management recommendations through health care providers.

## Introduction

Fasting in Ramadan is one of the five pillars of Islam [1]. Ramadan is the ninth lunar month of the Islamic calendar and its duration varies between 29 and 30 days [2]. All healthy adult Muslims are obligated to fast during Ramadan each year. However, if the health of the individual is adversely affected by fasting, Islam exempts that individual from holding fast for as many days as necessary [3].

During fasting, Muslims must abstain from taking food, water, beverages, smoking and oral medications from sunrise to sunset. However, there is no restriction on the intake of food or fluid between sunset and dawn [4]. The length of daylight hours and the duration of the fast vary considerably between summer and winter months, as Ramadan occurs 11 days earlier each year and hence every 9 years it occurs in a different season [5].

According to the 2009 estimates, Muslims constitute 1.57 billion of the world’s population and growing by 3% per year [6]. Globally, around 50 million Muslims with diabetes fast during the month of Ramadan each year [7]. The population based study, Epidemiology of Diabetes and Ramadan (EPIDIAR), highlighted certain important facts regarding fasting and diabetes management during Ramadan. The study was conducted in 12,243 patients with diabetes in 13 Islamic countries. It showed that 43% and 79% of patients with type 1 and type 2 diabetes reported fasting during Ramadan. Only 68% and 62% of the patients with type 1 and type 2 diabetes received Ramadan-specific diabetes management recommendations from health care providers. Blood glucose monitoring during Ramadan was done by only 67% and 37% of the patients with type 1 type 2 diabetes, respectively [1].

Interestingly, oral anti diabetic drug doses were continued unchanged in 79% and 75% patients with type 1 and type 2 diabetes, respectively. Whereas, insulin dose was continued unchanged in 64% of the patients with diabetes (both type 1 and type 2 diabetes). The frequency of at least one episode of severe hypoglycemia requiring hospitalization during Ramadan for patients with type 1 and type 2 diabetes was 9% and 2%, respectively. Similarly the frequency of at least one episode of severe hyperglycemia with/without ketoacidosis requiring hospitalization during Ramadan for patients with type 1 and type 2 diabetes was 13% and 4%, respectively. Physical activity, sleep duration, food intake, fluid intake and sugar intake during Ramadan were changed by only one half of the study population [1].

Based on these findings several recommendations were made by experts in the field of diabetes. Education of patients was identified as the cornerstone of safe fasting. Ramadan-specific education including drug dosage and timing alteration, blood glucose monitoring and dietary changes were identified as main factors to avoid acute complications of diabetes during Ramadan [1,3,4,8-11].

To the best of our knowledge no study was conducted to observe the implementation of these recommendations in a multi-centered setting. Hence, a multi-centered prospective study was designed. The aim of the study was to observe the outcome of the implementation of Ramadan-specific diabetes management recommendations in fasting individuals with diabetes through health care providers.

## Patients and methods

This prospective study was conducted at nine diabetes specialist centers in four provinces of Pakistan. The study was carried out in two phases; a pre-Ramadan recruitment interview (visit A) and a post-Ramadan follow up interview (visit B) of the same patients. Visit A commenced 15 days prior to Ramadan of 2011 (Hijri year, 1432) and continued until the first day of Ramadan, 2011. The second interview (visit B) was performed up to one month after the end of Ramadan, 2011. At each center; randomly selected health care providers (diabetologists or diabetes educators), involved in the care of patients with diabetes were assigned the task to enroll patients for the study after obtaining informed consent. Ethical approval for the study was obtained from the Institutional Review Board of Baqai Institute of Diabetology & Endocrinology (BIDE).

### Inclusion criteria

All subjects with type 1 or type 2 diabetes who gave history of fasting in the previous year and showed intention to fast in the coming Ramadan, were considered eligible to participate in the study.

### Exclusion criteria

Patients with type 1 or type 2 diabetes with serious complications, such as unstable angina, uncontrolled hypertension, severe liver or renal disease, newly diagnosed patients (< 3 month), pregnant women, patients with brittle type 1 diabetes, elderly patients with alertness problems, hospitalized individuals and patients with diabetes with hypoglycemia unawareness were excluded from the study.

### Patient counseling

The methodology of the study was explained and uniform recommendations and educational material regarding the information to be communicated to the patients at the time of recruitment in the study were presented to each health care provider (diabetologists or diabetes educators) before the start of the study. They were advised to assess the patient’s physical well-being and metabolic control at the time of recruitment in the study. An identical questionnaire was used at each center.

Pre-Ramadan individual counseling was given to each patient by the health care providers during visit A. The counseling session usually lasted for 15–20 min. Patients were educated regarding recognition of warning symptoms of possible complications such as hypoglycemia, hyperglycemia and dehydration. They were told to monitor blood glucose at home according to their usual routine and also when they experienced any hypo or hyperglycemic symptoms. Patients were advised to discontinue their fast immediately for the rest of the day in the case of any medical emergency and to seek medical care. Patients were also encouraged to continue their routine physical activities, although they were advised to avoid non routine heavy physical activity during fasting. Each patient was also provided written educational material regarding Ramadan-specific diabetes management recommendations.

The physicians (diabetologists) were recommended to adjust drug dosage and timing in fasting patients with diabetes. The advice to the patients was to take 75% of their full pre-Ramadan morning dose of oral hypoglycemic agents such as sulphonylureas, meglitinides as well as insulin, (alone or in combination) at Iftar (pre-dusk) and half of the pre-Ramadan evening dose at Sehar (pre-dawn). The rest of the oral hypoglycemic agents like metformin, thiazolidinedione and acarbose could be continued as their full pre-Ramadan morning dose of oral hypoglycemic agents taken at Iftar (pre-dusk) and half of the pre-Ramadan evening dose at Sehar (pre-dawn). However, the decision for altering the medication regimen was at the physician’s discretion, depending on the glycemic control of individual patients.

### Data collection

Two standardized questionnaire-based interviews were conducted by the physicians who enrolled the study subjects during face-to-face interviews.

### Pre-Ramadan interview (visit A)

In the visit A, detailed history was taken from the patient. Information collected included socio-demographic and anthropometric data, diabetes type, duration of the disease, comorbidities, frequency and type of current treatment of diabetes, diet, physical activity level including type of exercise and routine blood glucose monitoring practices. Laboratory investigations including fasting blood glucose, HbA1c and fasting lipid profile were also collected from the patient’s record (if available within 3 months).

### Post-Ramadan follow up interview (visit B)

In the visit B information collected included anthropometric data, number of days of fasting during Ramadan, development of symptoms of hypoglycemia and hyperglycemia and if developed, the frequency of the symptoms and the measures taken, hospital admission for any medical reason, frequency of blood glucose monitoring during Ramadan, frequency and type of treatment of diabetes during Ramadan and physical activity level including type of exercise during Ramadan.

### Symptomatic hypoglycemia

Symptomatic hypoglycemia was defined as the occurrence of one or few of the hypoglycemic symptoms such as palpitation, shivering, cold sweating, feelings of weakness, excessive hunger, visual problems, drowsiness and unconsciousness [12].

### Symptomatic hyperglycemia

Symptomatic hyperglycemia was defined as the occurrence of one or few of the hyperglycemic symptoms such as polyphagia, polydipsia, frequent urination, drowsiness and unconsciousness [12].

All the data was centralized into the computer data base of Baqai Institute of Diabetology & Endocrinology (BIDE). A total of 682 patients initially enrolled in the study (visit A). Of these 388 (56.89%) completed the follow up interview (visit B). Individuals with diabetes who did not attend the follow up interview (visit B) within the stipulated time period of one month after Ramadan were excluded from the final analysis.

### Statistical analysis

Data analysis was conducted on Statistical Package for Social Sciences (SPSS), version 13.0. All the continuous variables, i.e. age, duration of diabetes, waist circumference, weight, height, body mass index (BMI), systolic and diastolic blood pressure, fasting blood sugar, HbA1c, cholesterol, HDL, LDL and triglycerides, were presented as Mean±SD. Categorical variables, such as gender, education level, occupation, physical activity level and treatment modality were presented in the form of number and percentage. A paired *t*-test was utilized to find the difference in mean values and chi square was used for categorical variables. P<0.05 was considered statistically significant.

## Results

A total of 682 patients with diabetes (325 males and 357 females) attended the pre-Ramadan recruitment interview (visit A). Of these, there were 27 (3.95%) and 655 (96.04%) patients with type 1 and type 2 diabetes respectively. Table 1 shows the baseline demographic and biochemical characteristics of the studied population. Mean age of the patients with type 1 diabetes was 24.50±9.80 years whereas; patients with type 2 diabetes were older with the mean age of 53.24±10.73 years. Mean duration of diabetes in patients with type 1 and type 2 diabetes was 8.53±6.56 and 8.85±6.84 years respectively. Mean BMI was 22.02±4.13 kg ⁄m^2^ in patients with type 1 and 28.78±6.93 kg⁄m^2^ in patients with type 2 diabetes. Mean HbA1c was 9.69±2.37 and 8.67±1.87% in patients with type 1 and type 2 diabetes respectively. All the patients with type 1 diabetes were on insulin. Oral hypoglycemic agents was the treatment regimen in 52.40% patients with type 2 diabetes, whereas 30.30% patients were taking a combination of oral hypoglycemic agents and insulin and 17.30% patients were on insulin.

**Table 1:** Baseline demographic and biochemical characteristics of the studied population

	**Type 1 diabetes**	**Type 2 diabetes**	**Overall**
n	**27**	**655**	**682**
Male	12 (44.40%)	313 (47.80%)	325 (47.65%)
Female	15 (55.60%)	342 (52.20%)	357 (52.34%)
Age (years)	24.50±9.80	53.24±10.73	52.08±12.05
Duration of diabetes (years)	8.53±6.56	8.85±6.84	8.84±6.83
Waist circumference (cm)	78.18±8.31	97.28±11.62	96.49±12.11
Weight (kg)	57.35±11.42	72.42±13.18	71.83±13.44
Height (cm)	161.10±11.52	159.38±11.02	159.45±11.03
Body mass index (kg/m^2^)	22.02±4.13	28.78±6.93	28.17±5.65
**Education level**			
No formal education/primary education	6 (22.20%)	230 (35.20%)	236 (34.70%)
Secondary education	14 (51.90%)	212 (32.50%)	226 (33.23%)
Higher education/university level	7 (25.90%)	211 (32.30%)	218 (32.05%)
**Occupation**			
Manual labor	1 (3.80%)	70 (10.80%)	71 (10.55%)
Office employee	22 (84.60%)	482 (74.50%)	504 (74.88%)
Retired/unemployed	3 (11.50%)	95 (14.70%)	98 (14.56%)
**Physical activity**			
Sedentary/light activity	12 (66.70%)	282 (81.50%)	294 (80.77%)
Moderate physical activity	3 (16.70%)	61 (17.60%)	64 (17.58%)
Heavy physical activity	3 (16.70%)	3 (0.90%)	6 (1.65%)
**Type of treatment**			
Oral hypoglycemic agents	0 (0%)	339 (52.40%)	339 (50.29%)
Insulin	27 (100%)	112 (17.30%)	139 (20.62%)
Oral hypoglycemic agents+Insulin	0 (0%)	196 (30.30%)	196 (29.08%)
			
Systolic blood pressure (mmHg)	111.15±14.23	127.31±18.33	126.69±18.45
Diastolic blood pressure (mmHg)	75.93±7.34	79.55±10.24	79.41±10.16
Fasting blood sugar (mmoles/l)	9.24±4.02	8.59±3.02	8.57±3.08
HbA1c (mmol/mol)	82±2	71±-3	72±-3
HbA1c (%)	9.69±2.37	8.67±1.87	8.71±1.89
Fasting serum cholesterol (mmol/l)	4.13±0.8986	4.526±1.19	4.507±1.18
Fasting serum triglyceride (mmol/l)	0.98±0.715	1.83±1.43	1.79±1.422
Fasting serum HDL cholesterol (mmol/l)	1.068±0.136	1.046±0.402	1.047±0.39
Fasting serum LDL cholesterol (mmol/l)	2.36±0.928	2.65±1.01	2.64±1.01

Values are Mean±SD; n (%).

Waist circumference was found unchanged in 31.25% patients with type 1, increased in 43.75% and decreased in 25% of the patients with type 1 diabetes after Ramadan. In patients with type 2 diabetes, waist circumference was unchanged in 18.68%, increased in 39.93% and decreased in 41.39% of the patients. It was also observed that weight remained unchanged in 50% patients, increased in 20% and decreased in 30% of the patients with type 1 diabetes after Ramadan. In patients with type 2 diabetes weight remained unchanged in 34.78% patients, increased in 26.36% and decreased in 38.86% patients with type 2 diabetes after Ramadan.

However, no statistically significant difference (p>0.05) was observed in mean waist circumference, weight, BMI, systolic and diastolic blood pressures before and after Ramadan in patients with type 1 and type 2 diabetes, as shown in Table 2.

**Table 2:** Changes in waist, weight, body mass index and blood pressure before and after Ramadan (n=388)

	**Type 1 diabetes**			**Type 2 diabetes**			**Overall**		
	**Before Ramadan**	**After Ramadan**	**p-value**	**Before Ramadan**	**After Ramadan**	**p-value**	**Before Ramadan**	**After Ramadan**	**p-value**
Waist circumference (cm)	78.70±7.94	77.77±9.37	0.376	98.96±14.44	98.19±12.09	0.219	97.05±12.83	97.84±14.89	0.190
Weight (kg)	58.95±18.45	56.65±11.92	0.392	72.89±13.37	73.31±13.67	0.096	72.45±14.06	72.03±14.37	0.178
Body mass index (kg/m²)	24.16±10.18	21.80±3.70	0.192	28.91±5.91	28.93±5.94	0.835	28.65±6.30	28.54±6.06	0.400
Systolic blood pressure (mmHg)	107.36±14.84	108.42±13.85	0.749	126.59±16.96	127.81±17.56	0.155	126.87±17.88	125.65±17.35	0.144
Diastolic blood pressure (mmHg)	75.50±15.03	74.75±7.52	0.769	79.25±9.74	79.23±10.55	0.966	79.0±10.46	79.06±10.09	0.906

Values are Mean±SD. p<0.05 considered statistically significant.

In patients with type 1 diabetes drug dosage and timing were altered in 16 (80%) patients during Ramadan while, in 4 (20%) patients it was not altered (p<0.000). In patients with type 2 diabetes drug dosage and timing were changed in 325 (90.5%) patients, while it remained unchanged in 34 (9.5%) patients (p<0.000).

No significant change (p>0.05) in the level of physical activity was observed in patients with type 1 diabetes during Ramadan. However, in patients with type 2 diabetes, majority of the patients changed from moderate/severe levels of physical activity before Ramadan to light physical activity during Ramadan (p<0.000).

Table 3 shows the frequency of symptomatic hypoglycemia and hyperglycemia in the studied subjects during Ramadan. Overall, symptomatic hypoglycemia was observed in 92 (23.7%) patients; in 6 (35.29%) patients with type 1 and 86 (23.18%) patients with type 2 diabetes. Blood glucose level was checked by all the patients with type 1 and 71.43% patients with type 2 diabetes when they developed hypoglycemic symptoms during Ramadan. On the development of hypoglycemic symptoms, 33.33% patients with type 1 and 48% patients with type 2 diabetes discontinued their fast respectively. Similarly, overall hyperglycemic symptoms were felt by 59 (16.30%) patients; in 6 (33.33%) patients with type 1 and 53 (15.41%) patients with type 2 diabetes. All the patients with type 1 and 78% patients with type 2 diabetes checked their blood glucose level on the development of hyperglycemic symptoms. None of the patient with type 1 diabetes discontinued their fast when they felt hyperglycemic symptoms, while 18.87% patients with type 2 diabetes discontinued the fast on the development of hyperglycemic symptoms. None of the patients with type 1 and type 2 diabetes required hospitalization when they developed either symptomatic hypoglycemia or hyperglycemia. The mean blood glucose level at which patients with type 1 and type 2 diabetes discontinued their fast on the development of hypoglycemic symptoms was 3.33 mmol/l and 3.62±0.87 mmol/l respectively. Similarly, patients with type 2 diabetes also discontinued their fast on the development of hyperglycemic symptoms at mean blood glucose level of 17.05±3.49 mmol/l. None of the patients developed diabetic ketoacidosis and hyperglycemic hyperosmolar state.

**Table 3:** Frequency of symptomatic hypoglycemia and hyperglycemia during Ramadan

	**Type 1 diabetes**	**Type 2 diabetes**	**Overall**
**Symptomatic hypoglycemia (n=388)**			
No	11 (64.71%)	285 (76.82%)	296 (76.3%)
Yes	6 (35.29%)	86 (23.18%)	92 (23.7%)
**Checked blood glucose level if developed symptoms of hypoglycemia**			
No	0 (0%)	22 (28.57%)	22 (26.50%)
Yes	6 (100%)	55 (71.43%)	61 (73.49%)
**If developed symptoms of hypoglycemia**			
Continued fast	4 (66.67%)	38 (50.67%)	42 (51.85%)
Discontinued fast	2 (33.33%)	36 (48%)	38 (46.91%)
Visited doctor	0 (0%)	1 (1.33%)	1 (1.23%)
Needed hospitalization	0 (0%)	0 (0%)	0 (0%)
**Symptomatic hyperglycemia (n=362)**			
No	12 (66.67%)	291 (84.59%)	303 (83.70%)
Yes	6 (33.33%)	53 (15.41%)	59 (16.30%)
**Checked blood glucose level if developed symptoms of hyperglycemia**			
No	0 (0%)	11 (22%)	11 (19.64%)
Yes	6 (100%)	39 (78%)	45 (80.35%)
**If developed symptoms of hyperglycemia**			
Continued fast	6 (85.71%)	43 (81.13%)	49 (81.67%)
Discontinued fast	0 (0%)	10 (18.87%)	10 (16.67%)
Visited doctor	1 (14.29%)	0 (0%)	1 (1.67%)
Needed hospitalization	0 (0%)	0 (0%)	0 (0%)

Values are n (%).

## Discussion

In this study we observed that it is feasible to implement Ramadan-specific diabetes management recommendations in fasting individuals with diabetes. None of the fasting patients with type 1 and type 2 diabetes required hospitalization for any acute complications of diabetes during Ramadan.

Ramadan-specific patient education is considered a keystone for safe fasting in individuals with diabetes. In our previous study, we observed that with patient education, alteration of drug dosage and timing, dietary counseling and active glucose monitoring, the majority of the patients did not have any serious acute complications of diabetes during Ramadan [8]. Similar results were observed in the present study in a multi-centered setting.

Blood glucose monitoring during Ramadan is essential for patients with diabetes who hold fast during Ramadan and more particularly in patients with type 1 diabetes and also in patients with type 2 diabetes who require insulin [4]. Majority of the patients in the present study followed the advice of monitoring their blood glucose when they felt hypoglycemic or hyperglycemic symptoms during fasting.

It has been recommended that patients with diabetes should immediately discontinue their fast on the development of hypoglycemia (blood glucose of <3.3 mmol/l [60 mg/dl]) because their blood glucose may fall further if treatment is delayed. Moreover, fast should also be broken if blood glucose reaches <3.9 mmol/l [70 mg/dl] with in the first few hours of starting the fast [4]. If blood glucose level exceeds >16.7 mmol/l [300 mg/dl] fast should also be discontinued for the rest of the day [4]. In the present study, many individuals with diabetes followed the advice and broke their fast if blood glucose readings were either too low or very high which may have enabled them to prevent acute complications of diabetes during Ramadan.

Drug dosage and timing alteration during Ramadan is also suggested in studies and recommendations [4,8]. In this study, the advice was followed and drug dosage and timing were changed in the majority of the patients with type 1 and type 2 diabetes during Ramadan.

Studies have shown variable results regarding weight and BMI changes in patients with diabetes during Ramadan. In some studies patients had a reduction in weight and BMI during Ramadan [13,14]. However, no significant change in mean body weight and body mass index was observed in this study before and after Ramadan, similar to the findings of other studies [5,15,16]. This may be attributed to the positive effects of dietary and life style changes as advised during Ramadan.

Data regarding physical activities in fasting patients with diabetes during Ramadan is scarce. Studies have emphasized that light to moderate levels of physical activity on a regular basis during fasting is harmless for patients with type 2 diabetes [5]. Normal levels of physical activity may be continued. However, excessive physical activity should be avoided within the few hours of sunset meal as it may lead to an increased risk of hypoglycemia [4]. In this study majority of the patients with type 2 diabetes followed the advice and changed from moderate/severe levels of physical activity before Ramadan to light physical activity during Ramadan.

As fasting itself is a hypoglycemia prone condition, the prevalence of symptoms related to hypoglycemia was high (23.7%) in the present study, majority of these episodes were mild hypoglycemic episodes. However, none of the patients required hospitalization for symptomatic hypoglycemic episodes during Ramadan which may be the result of Pre - Ramadan patient education. The overall prevalence of symptomatic hyperglycemia in the present study was 16.30%, nearly identical to a retrospective study [12]. Although risk for hypoglycemia is the major concern for patients with diabetes during Ramadan, excessive consumption of sweet and fried food, especially at Iftar meal predispose the patients to hyperglycemia [17]. However, as a benefit of patient education regarding diet during Ramadan none of the patients in the study developed diabetic ketoacidosis and hyperglycemic hyperosmolar state.

Our study has some limitations; sample size was small and there was no control group i.e. individuals with diabetes without Ramadan-specific patient education. There was also no Pre and Post Ramadan data for comparison with the findings during Ramadan. Dropout rate in the study was high because individuals who did not attend the follow up interview (visit B) within the stipulated time period of one month after Ramadan were not included in the final analysis.

## Conclusion

We observed that it is practicable to implement Ramadan-specific diabetes management recommendations through health care providers. Patients with diabetes who intend to fast should undergo Pre-Ramadan assessment and receive appropriate highly individualized patient education. Health care providers should also be educated and trained to deliver appropriate advice in order to ensure safe fasting in patients with diabetes during Ramadan.

## Competing interests

The authors declare that they have no competing interests.

## Authors’ contributions

MYA Concept and design, edited and reviewed the manuscript, SFDA Researched data, wrote and reviewed the manuscript, MSU Researched data and reviewed the manuscript, AF Edited and reviewed the manuscript, AB Edited and reviewed the manuscript. All authors read and approved the final manuscript.
